# Relative Changes from Prior Reward Contingencies Can Constrain Brain Correlates of Outcome Monitoring

**DOI:** 10.1371/journal.pone.0066350

**Published:** 2013-06-20

**Authors:** Faisal Mushtaq, Gijsbert Stoet, Amy Rachel Bland, Alexandre Schaefer

**Affiliations:** 1 Institute of Psychological Sciences, University of Leeds, Leeds, United Kingdom; 2 School of Education, University of Glasgow, Glasgow, United Kingdom; 3 Neuroscience and Psychiatry Unit, University of Manchester, Manchester, United Kingdom; 4 Wolfson Research Institute, University of Durham, Thornaby, United Kingdom; University of Minnesota, United States of America

## Abstract

It is well-known that the affective value of an environment can be relative to whether it reflects an improvement or a worsening from a previous state. A potential explanation for this phenomenon suggests that relative changes from previous reward contingencies can constrain how brain monitoring systems form predictions about future events. In support of this idea, we found that changes *per se* relative to previous states of learned reward contingencies modulated the Feedback-Related Negativity (FRN), a human brain potential known to index discrepancies between predictions and affective outcomes. Specifically, we observed that environments with a 50% reward probability yielded different FRN patterns according to whether they reflected an improvement or a worsening from a previous environment. Further, we also found that this pattern of results was driven mainly by variations in the amplitude of ERPs to positive outcomes. Overall, these results suggest that relative changes in reward probability from previous learned environments can constrain how neural systems of outcome monitoring formulate predictions about the likelihood of future rewards and nonrewards.

## Introduction

After a long period of unemployment, the event of being offered a job with an average salary is almost always positively evaluated. This event can certainly lead to an upwards revision of a series of expectations (e.g. better house, better car, better holidays, family planning, etc.). In contrast, a high earner who has to change jobs and accept the same average salary job would instead have to revise downwards his/her expectations (e.g. smaller house, cheaper holidays, etc.). This trivial example illustrates a fundamental dimension of human cognition: the same objective event can be at the origin of radically different expectations if it represents a change from different prior circumstances. It is important to emphasize that the absolute value of the new situation (e.g. an average salary job) often has little relevance. What matters in this case is the *change* relative to a previous situation (an *improvement* or a *worsening* in overall expectations). This phenomenon is frequent in everyday life and can potentially be linked to psychopathological states linked to sudden changes in life circumstances [1]–[3].

A mechanistic explanation for this phenomenon could be inspired from reinforcement learning and conflict monitoring models [4], [5]. According to these models, the human brain keeps track of previous experiences (and in particular of instances of positive and negative reinforcements) in order to formulate predictions about incoming events. The accuracy of these predictions is measured by monitoring processes which are widely thought to rely on the medial frontal cortex, and in particular the Anterior Cingulate Cortex (ACC). If an event does not fit the predictions of the system, then a discrepancy signal, often called a reward prediction error (RPE), is produced and can be used to adjust future predictions [4], [6]. From this theoretical approach, it could be hypothesized that relative changes *per se* in the frequency of rewarding events could constrain how monitoring systems formulate predictions about incoming events. For instance, an environment characterised by a dynamic *increase* in reward probability relative to a previous environment could lead to the prediction that rewards will become more frequent. Consequently, the occurrence of nonrewards could reflect deviations from this expectation. Conversely, a relative *decrease* in rewards could lead to the expectation of a growing number of nonrewards, which would cause rewards to be perceived as prediction errors. Stated differently, relative changes from prior environments could determine how much an event deviates from ongoing predictions, above and beyond the absolute value of this event. These hypotheses would be consistent with theories emphasizing the role of contextual factors in the detection of prediction errors [7], [8]. For instance, Bar [7], [9] suggests that a context created by top-down memory representations can be the main determinant of whether an event is appraised as a prediction error, and consequently if this event will attract attentional resources or not. Applied to the case described above, it could be speculated that a change in reward probability could in itself form a context that determines if a (non)reward is a significant prediction error or not. These predictions would also be consistent with fMRI studies showing that medial frontal areas are sensitive to relative changes in reward contingencies (48–50) and with studies suggesting that RPE indices can be sensitive to context effects over and above absolute reward values [10], [11].

The Feedback-related Negativity (FRN) provides an opportunity to test these ideas. The FRN is a scalp event-related potential (ERP) time-locked to the delivery of decision outcomes, and it is characterised by a larger negative-going deflection for non-rewards compared to rewards [12]. Evidence indicates that the FRN is linked to activity in medial frontal areas, and in particular the ACC [13]–[15]. This distinction between ERPs to positive and negative outcomes is now perceived as reflecting a fundamental ability to differentiate between valenced outcomes and has become the object of intense investigation [12], [16]–[23]. An important characteristic of the FRN is its sensitivity to the unexpectedness of an outcome [21], [22]. In particular, many studies found that FRNs were larger for unexpected than expected negative outcomes (when outcomes are “worse than expected”) [24], [25]. Several studies show that this pattern is obtained for negative but not for positive outcomes, for which the effects of unexpectedness can often follow a different polarity (i.e. more ERP positivity for unexpected rewards in the FRN time window) [21], [22]. These results are consistent with the theoretical model most frequently used to explain FRN effects (the “reinforcement learning theory of the error related negativity”, or RL-ERN) which posits that the FRN is an index of negative RPE [4]. However, a growing number of studies indicate that the sensitivity of the FRN to unexpectedness does not always vary according to the valence of the outcome [26]–[30]. These results would be more consistent with a recent model that suggests that the FRN reflects the activity of a valence-independent system of detection of expectancy violations [31]. Despite this ongoing debate, there seems to be a consensus that the FRN indexes deviations from learned predictions [18].

**Figure 1:**
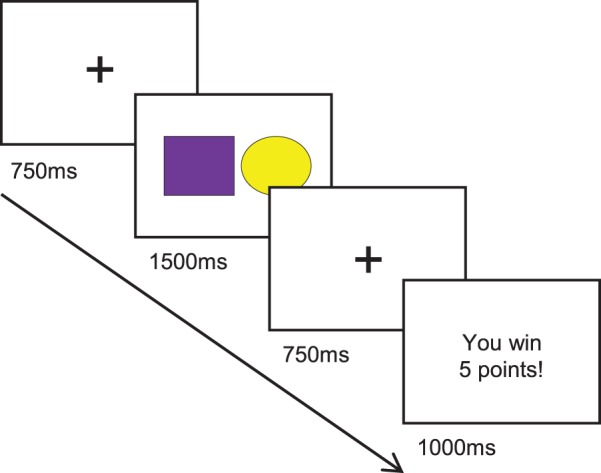
Task procedure. Each trial started with a fixation cross (750 ms) followed by the presentation of a screen including a circle and a square (one shape coloured yellow and the other purple) representing either a risky or safe choice. Participants made a selection between a risky and safe choice using a keypress. Next, another fixation screen was displayed and followed by a screen providing a feedback (1000 ms) detailing whether participants had won or lost points in the trial (see “Experimental task” for more details).

**Figure 2:**
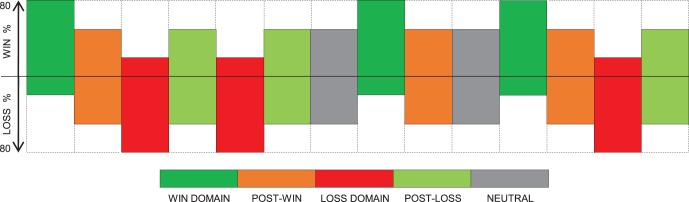
Example of a sequence of experimental blocks for one participant. In total, participants encountered 14 blocks with 32 trials each. The reward probability was manipulated within each block. The Win Domain (WD) block had a reward probability of 80% and the Loss Domain (LD) had a 20% reward probability. Blocks with a 50% reward probability followed WD and LD: Post-loss (PL) blocks always followed LD and Post-Win (PW) blocks always followed WD. The neutral blocks, which also had a reward probability of 50% did not represent a change in reward expectancy and were included in order to minimize the expectations that WD or LD blocks would necessarily appear after PW or PL blocks. A randomised sequence of three WD-PW block pairs, three LD-PL block pairs and two neutral blocks were presented to each participant.

Therefore, the main goal of the present study was to use the FRN in order to examine whether RPEs can be constrained by a change *per se* relative to a previous state of reward contingencies. Specifically, we predicted that environments with a constant 50% reward probability would yield different FRN patterns according to whether these environments correspond to an increase or a decrease in reward probability relative to a previous context. To test this hypothesis, this study used a gambling task where participants were delivered a series of feedbacks reflecting financial gains or losses (see Figure 1). More specifically, participants had to perform a forced two-choice decision task followed by a financial gain or loss over the course of several trials. The experimental trials were divided in four blocked conditions (each containing 32 trials) across which the reward probability was manipulated: the “Win Domain” (WD), the “Loss Domain” (LD), the “Post-Win Domain” (PW) and the “Post-Loss Domain” (PL). In WD, 80% of the trials led to a financial gain. In LD, 80% of the trials led to a financial loss. Crucially, these two conditions were each followed by a context in which the relative proportions of gains and losses were equal (50%): the PL and the PW blocks (more specific details are described in the Procedure section, and in Figures 1 and 2). We hypothesized that WD and PL would both be characterized by *positive* expectations. In other words, WD and PL would be contexts in which rewards are expected, and nonrewards are unexpected. These putative expectations would be induced either in an “objective” manner for WD (i.e. through the manipulation of reward/nonreward frequencies) or “subjective” in the case of PL (i.e. through the *improvement* in reward probability relative to the previous block). Conversely, we expected LD and PW to be contexts characterized by *negative* expectations induced either by a low frequency of rewards (LD) or by a *worsening* relative to the previous block (PW).

Given that many FRN studies seem to report a valence asymmetry in FRN effects consistent with the RL-ERN account (i.e. the FRN is larger when outcomes are *worse* than expected; see [21] for a review), we predicted that the effect of valence on the FRN should be reliable when expectations are positive (negative feedbacks are unexpected) and it should be smaller or null when expectations are negative [32], [33]. Therefore, the effect of valence on the FRN should be robust for both WD and PL, but smaller or null for LD and PW. Crucially, if a change *per se* relative to a previous state of reward probability is sufficient to modulate outcome monitoring systems, then there should be no difference between expectations induced by “objective” vs. “subjective” methods. Specifically, the behaviour of the FRN should be similar between WD and PL, and between LD and PW. Finally, the gambling task used in the present study also required participants to choose between two possible options: a “risky” option for which the magnitude of both positive and negative outcomes was in average larger than a “safe” option (see Procedure and Design section). This approach enabled us to control for the type of behavioural choice preceding the outcome, a variable that can sometimes modulate the FRN [34] and which is often not explicitly controlled for.

## Materials and Methods

### Participants

Thirty right-handed healthy participants (17 males; mean age = 22.87 years, SD = 4.67) with normal or corrected-to-normal vision and with no history of psychiatric or neurological conditions participated in this study. Six participants were excluded because of excessive EEG artifacts leading to less than 16 artifact-free trials for at least one of the relevant experimental conditions. Two participants were removed because they were behavioural outliers. Behavioural outliers were defined as participants who displayed choice behaviour rates that were more than 1.5 interquartile ranges below the lower quartile in any relevant experimental block, as recommended by Tukey [35]. All analyses were performed on the resulting sample of 22 participants (12 males, mean age = 23.68 years, SD = 5.2). All participants signed an informed consent and the study was approved by the Ethics Committee of the Institute of Psychological Sciences at the University of Leeds.

### Procedure and Design

The experiment took place in a quiet room with lights dimmed. After the setup of the EEG electrode net, participants were invited to sit comfortably at approximately 50 cm away from a computer screen and were instructed to position their right hand on a stimulus response pad (Psychology Software Tools Serial Response Box, Pittsburgh, PA). The experiment was displayed on a 17″ Dell monitor, with a screen resolution of 1280 × 1024 and refresh rate of 60 Hz, and controlled by E-Prime (Psychology Software Tools, Pittsburgh, PA). Prior to the experiment, participants were told that they would take part in a gambling experiment in which they could successively choose a “risky” or a “safe” choice that would be followed by gains or losses of points relative to an initial lump amount of 1000 points. They were also told that at the end of the experiment, the final amount of points would be translated into an actual sum of money of up to £10. As depicted in Figure 1, on each trial, participants were first shown a fixation cross during 750 ms. Next, participants were shown a screen displaying two shapes; a circle and square (1500 ms), with one shape coloured yellow and the other purple. Each of the two colours was linked to either a risky or a safe choice. Participants were explicitly told before the experiment which of the two colours was linked to a risky or safe choice. The association between coloured shapes and response type (risky vs. safe) was counterbalanced across participants. Choosing a risky option would lead to a relatively large amount of points (a randomised amount between 5 and 9 points) gained or lost, whereas a safe choice would lead to a relatively low amount (a randomised amount of points between 1 and 4) of points won or lost. As soon as the coloured shapes appeared on screen, participants had to choose between these two options with a keypress. In order to minimise strategic no-responses, if no key was pressed 1500 ms after the onset of the screen presenting coloured shapes, a randomised amount between 1 and 9 points was deducted from the total score. After choice selection, a fixation cross (750 ms) preceded the feedback presentation stimuli, which appeared on the screen for 1000 ms. The feedback screen provided information about the valence of the feedback (“You Win!” or “You Lose!”), a plus or minus signal to indicate reward or punishment and the amount of points to be added or subtracted from the total score.

Each participant was presented with a total of 448 trials. These trials were separated into 14 blocks, each containing 32 trials for an experiment that lasted approximately 70 minutes. As explained in the introduction,there were four main types of blocks: Win Domain (WD), Loss Domain (LD), Post-Win domain (PW) and Post-Loss domain (PL). In WD, most of the outcomes (80%) reflected financial gains and in LD, most of the outcomes (80%) were losses. These two contexts were each followed by a context in which the relative proportions of gains and losses were equal (50%): the PL and the PW domain. In addition to these four block types, “Neutral” blocks appeared twice in the experiment after a 50% reward probability block. The Neutral blocks also had a 50% reward probability and were included in order to minimize the expectations that win or loss block would necessarily appear after a PW or PL block. Each participant was therefore presented with a randomised sequence of three WD-PW block pairs, three LD-PL block pairs and two neutral blocks, for a total of 14 blocks with 32 trials for each block. Before the experiment, participants performed 8 practice trials in order to familiarize with the procedure.

### Electrophysiological Data Recording and Analysis

**Figure 3:**
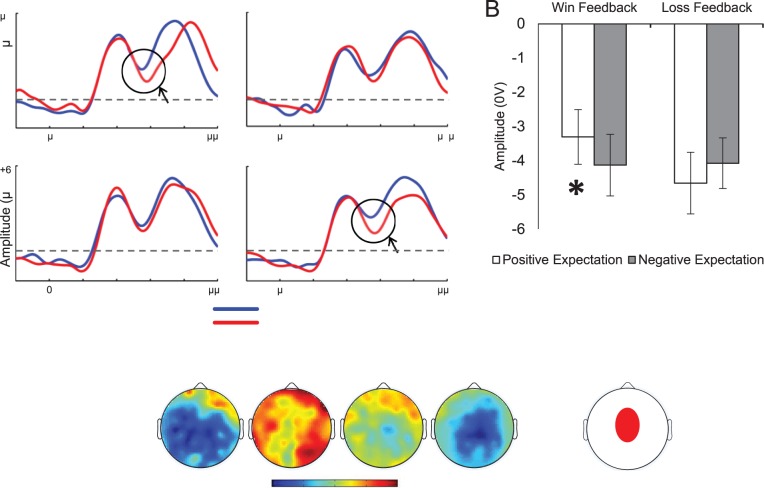
FRN data. **Figure 3a** **.** Averaged ERP waveforms for the Feedback-Related Negativity (FRN) plotting ERPs to Wins and Losses separately for the four experimental conditions. A 1–12 Hz bandwith was applied for presentational purposes. **Figure 3b** **.** Valence-specific comparisons of peak-to-peak amplitudes (in µV) between positive and negative expectancy contexts, separately for ERPs to Wins and Losses. Error bars represent one standard error from the mean. * *p*-value<.05. **Figure 3c** **.** Topographical maps plotting difference scores between Loss minus Win ERPs (peak to peak amplitudes) between 250 and 350 ms (maxima: +1.0 µV, minima: −3.5 µV). **Figure 3d** **.** Scalp location of cluster of electrodes used to quantify the FRN.

**Figure 4:**
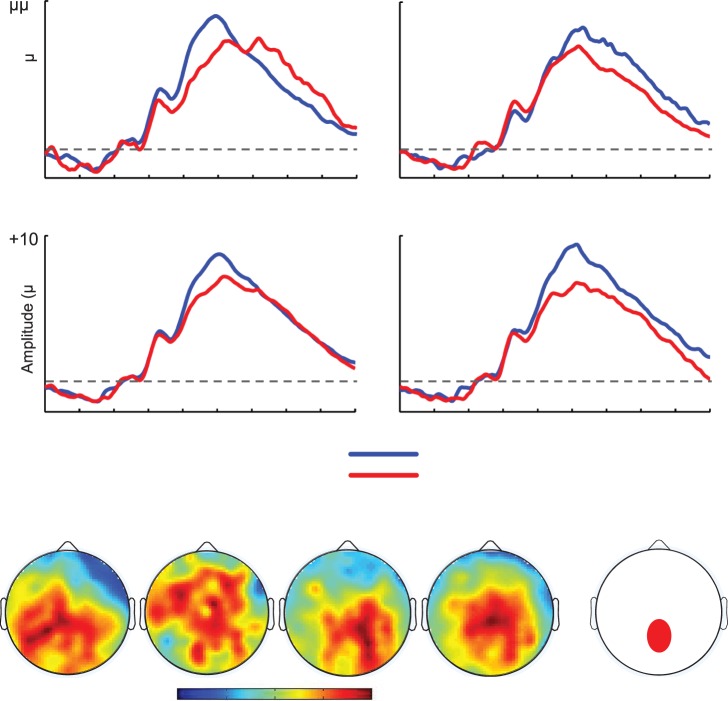
Neutral condition ERPs. Averaged ERP waveforms for the Feedback-Related Negativity (FRN) plotting ERPs to Wins and Losses in the neutral blocks.

EEG was recorded with a 128-channel net connected to a high-input amplifier (Electrical Geodesics, Inc., Eugene, OR) at a rate of 500 Hz (0.01–200 Hz bandwidth). All electrodes had an impedance below 50 kΩ. However, the impedance was lowered at or below 20 kΩ for those electrode sites likely to be included in our analyses (including all the electrodes belonging to the clusters described in Figure 3d and 4c). EEG data were recorded using a Cz reference, and digitally converted to an average mastoids reference. EEG data were analysed using the ERP module of BESA 5.1 (MEGIS Software GmbH, Gräfelfing, Germany). EEG data were further filtered offline (0.1–30 Hz bandwith) and segmented into epochs of 0–1000 ms time-locked to the onset of the “win” or “lose” feedback (with an additional 200 ms pre-stimulus baseline). Eye movement artifacts were corrected using a multiple source analysis method [36], [37] as implemented in BESA 5.1 (“surrogate method”). In addition, for each channel, epochs with a difference between the maximum and minimum voltage amplitude>120 µV and a maximum difference between two adjacent voltage points>75 µV were rejected (after eye movement artifact correction). ERP waveforms were first created through averaging baseline-corrected EEG data epochs for eight trial types corresponding to “Win” and “Lose” feedbacks for the 4 block types (WD, LD, PW, PL). An average of 44 artifact-free trials by condition was attained, and participants with less than 16 artifact-free trials in any relevant condition were excluded from the sample (see Participants section).

Following our hypotheses, data analyses focused mainly on the Feedback-related Negativity (FRN). As the FRN is usually observed mainly in midline fronto-central sites [10], [38], we focused on this location for our analyses, following standard practice [12], [16], [26], [28], [39]. We formed a cluster in which we averaged electrode data from a group of midline electrodes surrounding the standard FCz location (EGI electrode numbers: ‘12′, ‘5′, ‘6′, ‘13′, ‘112′, ‘7′, ‘106′, ‘Cz′, ‘31′, ‘80′ and ‘55′, see Figure 3d). This approach is consistent with common practice in high-density EEG research according to which pooling single electrode data in clusters improves the stability of ERP data and attenuates familywise statistical errors [40]. Analyses on the FRN focused on negative peak amplitudes extracted from the fronto-central cluster in a 250–350 ms time window, consistent with previous literature [41]–[43]. In order to further verify the robustness of our FRN results, we also computed our analyses on mean amplitudes and peak-to-peak amplitudes. Peak-to-peak amplitudes were computed by subtracting the positive peak in the 150–250 ms time window from the negative peak in the 250–350 ms window. In order to check if our results were not due to the utilization of a cluster of electrodes rather than single electrodes, we verified that similar results were obtained using a single electrode approach which is often used in FRN studies.In particular, we observed a similar Feedback X EVal interaction (*p* = .006) on FRN data using single electrode e6, which approximates the FCz standard location.

In addition to the FRN, we examined the Feedback-related P3, a component also known to be sensitive to decision outcomes. Its precise functional meaning is still unclear, but evidence suggests that it may be sensitive to outcome valence, magnitude and expectancy [12], [26], [44], [45]. We did not have *a priori* hypotheses regarding this component, but we also examined it in order to allow comparisons with previous research. As the Feedback-related P3 is usually measured in posterior sites [12], we created a midline parietal cluster surrounding the standard Pz location (EGI electrode numbers: '61′, ‘78′, ‘62′, ‘67′,‘72′,‘77′, ‘71′ and ‘76′, see Figure 4c). Given that amplitude differences at the onset of the P3 (around the N200) were visible, absolute peak and mean amplitudes might have been biased and thus we focused only on peak-to-peak measures. Peak-to-peak amplitudes were obtained by subtracting negative peak amplitudes from a 250–350 ms time window from the positive peak amplitude obtained from the 350–500 ms time window.

The choice of the electrode locations and time windows for both the FRN and Feedback-related P3 was guided by previous literature [16], [18], [38], [46], [47] and by a careful inspection of our waveforms. A Feedback (win vs. loss) X EVal (Expectation valence: positive vs. negative) X EType (Expectation type: subjective vs. objective) repeated measures ANOVA was computed for both the FRN and the P3. A “positive” expectation valence referred to contexts in which we assumed that positive outcomes would be expected, and negative outcomes unexpected (WD and PL). “Negative” expectation contexts would have the opposite characteristics (LD and PW). “Objective” expectations refer to manipulations of reward frequencies in the current block (WD and LD) whereas “subjective” expectations refer to manipulations relative to the previous block (PL and PW). Given that our hypotheses focused on the well known electrophysiological distinction between negative and positive feedback, we report only statistical effects involving the Feedback factor. Significant interaction terms were followed up by pairwise comparisons between Win and Loss ERPs. ANOVAs were computed with the Greenhouse-Geisser correction where relevant to ensure that results are not biased by potential violations of sphericity. We consider statistical effects to be reliable at *p*≤0.05, and we also report the partial eta-squared measure of effect size where relevant.

We also performed a number of additional analyses: first, we examined whether the type of behavioural choice (risky vs. safe) had any effect on the FRN and P3. Second, we examined if our effects on the FRN were modulated by individual differences in risk-taking. Third, we examined if our results were different according to different temporal stages within each block of trials. The outcomes of these analyses did not modify the main conclusions of this study. However, for completeness, we report these analyses in detail in the supplementary sections (specifically, see Results S2, Figure S1, Figure S2, Results S3, Table S3, Figure S3, Figure S4; Results S4, Figure S5 and Figure S6).

## Results

### Behavioural Results

Given that behavioural responses (risky vs. safe choices) were not predictive of feedback outcome, the type of behavioural choice preceding feedback cannot be considered as a meaningful behavioural correlate of the FRN. We have nonetheless analyzed this behavioural data for the sake of completeness and in order to assess whether the context manipulation had affected the type of behavioural choice (risky vs. safe). We analysed response frequency and response time (RT) using an EVal X EType X Choice repeated measures ANOVA. The RT data revealed a marginally significant main effect of EVal (*F*(1,21) = 4.1, *p = *.056, *η* ^2^ = .16), reflecting faster responses for negative expectancy valence blocks (*M* = 547, *SE* = 23.7) compared to positive expectancy valence blocks (*M* = 562, *SE* = 24.7). This is consistent with previous research demonstrating faster responses for aversive stimuli [48] and negative affective states [49], [50]. No other significant effect was found. Analyses on choice frequency revealed no significant variations in the choice of risky/safe options across blocks. Specifically, there was no effect of Choice (*F*(1,21) = 1.2, *p = *.285, *η* ^2^ = .05), no EType X Choice interaction (*F*(1,21) = 1.5, *p = *.23, *η* ^2^ = .07), no EVal x Choice (*F*(1,21) = 1.0, *p = *.32, *η* ^2^ = .05) and no EVal X EType X Choice (*F*(1,21) = .11, *p = *.74, *η* ^2^ = .005) interactions. Descriptive statistics of these analyses can be found in Results S1, Table S1 and Table S2.

### FRN

Consistent with our predictions, a sizeable differentiation between ERPs to wins and losses was found in WD and PL, but not in LD and PW blocks (see Figure 3a). Consistent with previous research [25], scalp maps show that the distribution of this effect is widely distributed across the scalp and clearly includes fronto-central sites (Figure 3c). Statistical analyses on peak amplitudes obtained from the Fronto-Central cluster revealed a robust EVal X Feedback interaction (*F*(1, 21) = 9.0, *p = *.007, *η* ^2^ = .30). This interaction was driven by a significant effect of Feedback for positive expectations (*F*(1, 21) = 8.5, *p = *.008, *η* ^2^ = .29), while this effect was not significant for negative expectations (*F*<1). The EVal X Feedback X EType interaction was not significant (*F*<1), indicating that the interaction between valence and expectation was not modulated by how expectations were manipulated (objective or subjective). We repeated this analysis using mean amplitudes and peak-to-peak measures, and the same EVal X Feedback interaction was found for both measures (*F*s>4.8, *p*s<.04), and these interactions were also driven by an effect of Feedback in positive expectation contexts specifically (*p*s<.003). In these cases, EType also failed to modulate the Feedback X Expectation interaction. In summary, these results indicate that a robust electrophysiological differentiation between rewards and nonrewards was observed in contexts thought to convey positive expectations (WD and PL) but not in contexts characterised by negative expectations (LD and PW). In addition, these findings did not differ according to the method used to induce expectations (manipulation of the reward probability of the current block or a change relative to the previous block).

Although the classical FRN effect involves assessing the difference in amplitude between ERPs to negative and positive feedbacks, a growing number of studies have recently suggested that variations in FRN effects might often be driven by differences specific to ERPs to reward related feedback [28], [29], [51]–[54]. In order to examine this question in our data, we re-analyzed our data breaking down the reported EVal X Feedback interaction by feedback type (Win or Loss). For peak amplitudes, we found a highly significant effect of EVal (*F*(1, 21) = 15.4, *p = *.007, *η* ^2^ = .42) for Win ERPs, but not for Loss ERPs (*F<*1). This effect appears to have been driven by a larger negativity of Win ERPs in negative expectation contexts. The same pattern was also obtained with mean amplitudes and peak-to-peak amplitudes, where we found a significant effect of EVal for Win feedbacks in both measures (*F*s>5.9, *p*s<.02), whereas this effect was not significant for Loss feedbacks (*F*s<3.2, *p*s>.09). Although descriptive statistics from peak-to-peak analyses suggest that FRNs to negative outcomes appear to be larger in positive compared to negative expectation contexts, we found that this difference was not formally reliable (*p = *.09), whereas the converse effect (larger FRNs for Win ERPs in negative expectation contexts) was clearly significant (*p = *.02). A more fine-grained block-by-block examination of these results suggests that win ERPs were sensitive to both WD-LD and PL-PW contrasts whereas Loss ERPs were sensitive to WD-LD but not PL-PW (see Figure S7). Relevant descriptive statistics are depicted in Figure 3b and Figure S7. Overall, these results suggest that variations in ERPs to positive feedbacks have played a predominant role in our FRN results.

The significant modulation of ERPs to positive outcomes suggests that an enhancement of FRN negativity for unexpected positive outcomes might have attenuated the difference between ERPs to negative and positive outcomes in PW and LD, which could have contributed to the EVal X Feedback interaction. In other words, the overall pattern of FRN results might have been driven by an attenuation of Win-Loss FRN differences in negative contexts, rather than by an enhancement in positive contexts. In order to examine this question, we considered neutral blocks as a baseline condition. Neutral blocks had a 50% reward probability that did not reflect a change from a previous block as they were always following blocks with similar reward probabilities (PW and PL). They had been inserted purely to attenuate expectations about what blocks would follow PL and PW blocks and thus were not initially included in the data analysis design. However, they can be seen as a “baseline” block in which no *a priori* expectations can be assumed. If an attenuation of FRN effects has taken place in negative contexts, then the Loss-Win difference in the FRN time window should be smaller in negative expectation contexts compared to both positive expectation and neutral baseline blocks.

In order to test this prediction, we calculated peak-to-peak FRN difference scores (Loss ERPs minus Win ERPs) for positive, negative and neutral contexts and computed a one-way ANOVA testing the effect of context (positive vs. negative vs. neutral) on these difference scores. We found a significant main effect of context (*F*(1.9,40.1) = 3.6, *p = *.04, *η* ^2^ = .14), and pairwise comparisons revealed that the FRN difference score for negative contexts was significantly smaller than both positive and neutral contexts (*p*s<.05). In addition, no significant difference was found between positive and neutral contexts (*p = *.90). Descriptive statistics for this analysis are included in Figure S8. In order to further verify the reliability of these effects, we observed that the effect of valence on FRN activity was significant in neutral blocks using peak amplitudes (*F*(1,21) = 8.5, *p = *.008, *η* ^2^ = .29). The same effect was obtained using peak to peak and mean amplitudes (*F*s>6.4, *p*s<.009). This effect is depicted in Figure 4. Finally, a Block (WD vs. LD vs. PL vs. PW vs. Neutral) vs. Feedback (win vs. loss) ANOVA revealed an interaction (*F*(2.3,47.9) = 3.4, *p = *.03, *η* ^2^ = .14) in the fronto-central cluster in which only PW and LD had clearly non-significant effects of feedback (*F*s<1.1, *p*s>.31). These findings suggest that our results were driven at least in part by an attenuation of the effect of valence on the FRN in negative expectation contexts, caused by a modulation of ERPs to positive outcomes in these contexts.

### P3

**Figure 5:**
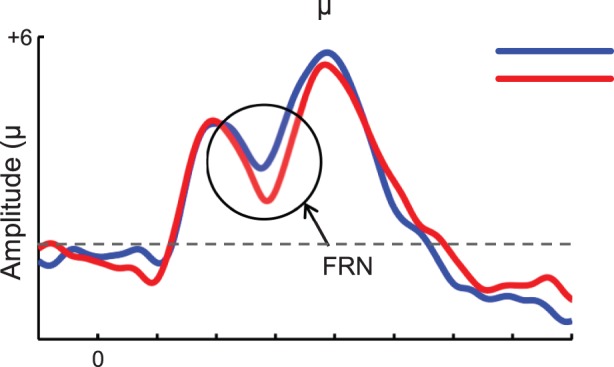
P3 data. **Figure 5a**. Averaged ERP waveforms for the Feedback-related P3 plotting ERPs to Wins and Losses separately for the four experimental conditions. **Figure 5b** **.** Topographical maps plotting difference scores between peak-to-peak amplitudes of Win minus Loss ERPs between 250 and 350 ms (maxima: +1.0 µV, minima:−3.5 µV). **Figure 5c** **.** Scalp location of cluster of electrodes used to quantify the FRN.

As shown in Figure 5, a larger positive peak is visible for Win ERPs compared to Loss ERPs. Given that visible differences around N200 could have driven onset differences biasing absolute peak and mean amplitude measures of the P3, we focused these analyses on peak-to-peak measures (see Methods section). We found a significant main effect of Feedback (*F*(1,21) = 5.4, *p = *.03, *η* ^2^ = .20) showing that positive outcomes yielded overall larger peaks than negative feedbacks, confirming our observations. We also observed a complex 3-way interaction between Feedback, EVal and EType (*F*(1,21) = 5.9, *p = *.02, *η* ^2^ = .22). Subsidiary analyses showed that this effect was driven by a Feedback X EType interaction in negative expectation contexts (*F*(1,21) = 4.4, *p = *.05, *η* ^2^ = .17), indicating that the Feedback effect was statistically more reliable in LD (F(1,21) = 7.1, *p = *.01, *η* ^2^ = .25) than in PW (*F*<1). Valence-specific analyses did not yield significant effects of interest.

## Discussion

The main finding of this study is that a change *per* se in reward probability relative to a previous environment can modulate the Feedback-Related negativity. Specifically, a reliable distinction between ERPs to negative and positive feedbacks in the FRN time window was observed in the WD and PL conditions, but not in LD and PW. This finding indicates that the evaluation of outcomes for a given reward probability (50% in the cases of PL and PW) can be different according to whether this probability reflects an increase or a decrease in reward probability relative to a prior context. In addition, valence-specific analyses suggested that variations in ERPs to positive feedbacks have played a predominant role in these effects. Further, we observed an overall effect of outcome valence on the P3.

The key finding of this study is that a mere increase or decrease in reward probability relative to a previous environment led to the same pattern of FRN modulation as a manipulation of the actual frequencies of rewards in the current environment. This finding is consistent with our hypothesis that a relative change *per se* from previous reward contingencies can constrain outcome monitoring systems. This implication is supported by a vast body of research indicating that the FRN is a neural index of outcome monitoring systems [12], [16] and by evidence indicating that the FRN is an index of discrepancies between predictions and valenced outcomes [25], [55], [56]. These findings are also consistent with previous data showing that the FRN is sensitive to context manipulations [10], [55]. However, these studies had manipulated context through the variation of the range of possible outcomes in a given environment, or by the manipulation of beliefs about future outcomes. The sensitivity of the FRN to changes *per se* (improvement or worsening) relative to prior states of learned reward contingencies remains largely unexplored.

Previous fMRI and animal model studies had shown that changes relative to previous states of reward probability could modulate how medial frontal systems respond to rewards and punishments [57]–[59]. However, our findings that similar manipulations can affect the FRN suggest that these effects can potentially be explained by an account related to the monitoring of deviations from reward expectancies. Specifically, our findings suggest that movements upwards or downwards in reward probability can be coded by brain monitoring systems and lead to a “resetting” of predictions about what future outcomes are the most and least likely to occur. Stated differently, a change per se in reward probability can create a *context* that determines what outcomes will be perceived as violations of established predictions [7]. Specifically, the context in this case could be a representation that the reward probability is increasing or decreasing, which in turn determines which type of outcome is expected to occur most frequently. Further research will be needed to investigate the precise brain structures involved in the putative re-setting of outcome predictions suggested by our findings. A significant amount of evidence indicates a link between the Anterior Cingulate Cortex (ACC) and the FRN [4], [16], [60] and between the ACC and the monitoring of outcome-prediction discrepancies [6], [61], [62]. However, fMRI studies that investigated relative changes in reward environments revealed that a wider network was involved in such effects, including ACC structures but also the orbitofrontal cortex (OFC) and subcortical areas [57]–[59]. In addition, recent research suggests that the insula may also be important for the detection of deviations from predictions, and for how subsequent learning can be guided by these prediction errors [8], [63]. Future research will be needed to investigate whether the effects of relative context changes on these areas can be accounted for by differences in outcome prediction setting.

The present findings also provide evidence that can contribute to a better understanding of the relationship between the FRN and reward environments. The FRN is classically operationalized as a difference between ERPs to negative and positive outcomes in the N2 time window [64]. Our findings that this difference between rewards and nonrewards is most reliable in “positive” expectation contexts seems consistent with the RL-ERN model according to which the FRN reflects a negative RPE [4], [24]. However, this conclusion needs to be considered within the context of two additional findings in our data. First, we found that the the interaction between Feedback and Expectancy valence in our data was mainly due to variations in ERPs to rewards rather than nonrewards. This finding is consistent with previous studies showing that FRN effects might be more related to neural activity related to rewards rather than nonrewards [51], [52], [64]. Second, we found that ERPs in the FRN time window were more negative-going for unexpected positive outcomes (in blocks LD and PW) compared to expected positive outcomes (in blocks WD and PL). This finding does not fit with the RL-ERN account, whereas it is consistent with the Predicted Response-Outcome (PRO) model (6). The PRO model suggests that the FRN reflects the activity of a system of detection of expectancy deviations, regardless of whether these deviations are positive or negative [31]. It has to be acknowledged that existing evidence regarding the modulation of the FRN by positive feedbacks is contradictory. Several studies report a reduced FRN (i.e. a larger ERP positivity in the FRN time window) for unexpected rewards (see [21] for a list of studies showing this result), whereas other studies obtain results similar to ours [26]–[30]. There is to our knowledge no consensual explanation that reconciles these contradictions, although several lines of thinking have been put forward: First, it is possible that ERPs to positive feedbacks in the FRN time window might reflect the overlap of different ERP components [64]. Second, it has been suggested that the motivational relevance of unexpected outcomes might, in certain paradigms, be different according to the valence of the outcome [28]. Therefore the salience of unexpected outcomes might be different according to outcome valence, which could explain why in many cases there is a valence X unexpectedness interaction in FRN data. Third, it has also been suggested that these contradictions might be due to systematic methodological biases in the FRN literature [26]. More research is necessary to explore these possibilities and resolve current contradictions in the FRN literature.

Although we had no specific hypotheses about the Feedback-related P3, we also examined this component to allow comparisons with previous studies. We observed an overall effect of valence in which ERPs to positive outcomes were more positive-going than ERPs to negative outcomes. Further, a three-way interaction on peak-to-peak measures suggested that this effect was more statistically reliable in LD compared to PW blocks. The larger positivities for Win rather than Loss feedbacks are consistent with many previous studies [42], [55], [65]. The observation that this effect was statistically stronger in LD than PW blocks could potentially be consistent with previous reports that the P3 might be sensitive to positive RPEs [66]. Both LD and PW are thought to be environments where positive outcomes are unexpected, but positive outcomes are more infrequent in LD than PW. Therefore, it could also be tentatively inferred that the P3 is not sensitive to relative contextual changes in reward probability. However, this conclusion has to be drawn with caution, given that the literature on the Feedback-related P3 has yielded many contradictory results so far. For instance, some studies have found a more positive peak for positive compared to negative feedbacks [67], [68], whereas other studies have found the opposite pattern [34], [45], [69], [70], or have found that valence does not modulate this component [12], [65]. Next, evidence that the P3 is sensitive to unexpected positive outcomes has been reported [71], [72], but it has also been shown that the P3 is sensitive to unexpected outcomes independently of valence effects [55], [71]. Finally, a few studies have reported that the effect of valence on the P3 was most reliable when certain types of outcomes were expected [42].

A potential explanation for these apparent contradictions is that the Feedback-related P3 is modulated by multiple factors that have yet to be thoroughly disentangled. In particular, the P300 complex is known to be sensitive to attentional factors [73] that could be intrinsically embedded in the paradigms used to investigate ERP correlates of reward outcomes. For instance, attentional parameters at feedback delivery could potentially be determined by variations in the type of choice, uncertainty and task demands of the behavioural choice that precedes the feedback [45], [74]. It could also be linked to the specific expectations created by the environment when feedbacks are delivered [66]. Future research programmes will be necessary to better understand and control attentional parameters involved in outcome monitoring to help resolve current contradictions.

Finally, the findings reported in the present paper can provide additional suggestions for future research. First, we observed a cancellation of the effect of valence on FRN activity in PW and LD blocks, which could appear surprising given the robustness of Win-Loss FRN differences in the literature. However, the absence of a differentiation between gains and losses is not uncommon in the literature [34], [75]. The reasons that might lead to a cancellation rather than an attenuation of the FRN effect are not easy to delineate, but at least two explanations are possible: (1) In our experiment, expectations were built up and consolidated over a relatively sustained period of time (i.e. over many trials). Therefore it is possible that nonrewards might have completely lost any meaning of unexpectedness/prediction error. In other words, building expectations over a sustained sequence of trials could lead to very stable negative predictions. In such a scenario, negative outcomes are unlikely to produce an RPE strong enough to generate an FRN effect. (2) Another explanation is linked to our findings that Win waveforms are strongly modulated by our manipulation. In our data, Win waveforms tend to be more negative-going when they are unexpected [27], [28]. Therefore win ERPs tend to go down to the same level of the Loss waveforms in LD and PW, which could explain the final result of an absence of Win-loss differentiation in those conditions. Future research will be needed to investigate these ideas, and more specifically (1) the effects of the stability of learned expectations on FRN activity; and (2) the role of ERPs to positive outcomes in general FRN effects. Second, we found no evidence that our effects were different according to whether the first or the second half of each block was considered (see Results S4 and Figure S5). However, it is not excluded that temporal differences might exist between shorter temporal stages within each block. Such a detailed analysis was beyond the scope of the current project, and not enough artifact-free trials were available to follow this approach in the current dataset. A possible methodological approach for future studies that might want to explore the temporal modulation of FRN activity within a block of trials could adopt a design with a substantially larger number of blocks. This approach could allow one to obtain enough artifact-free trials for a detailed separation of blocks into very short temporal stages, although the effects of an overly long experimental session on the results should be carefully considered. Third, our findings show that a 50% reward probability can lead to different FRN patterns according to the reward probability encountered before the current environment. However, future research will be needed to explore the extent to which this phenomenon is independent from the absolute values of reward probabilities involved. For instance, it remains to be seen if a similar modulation of the FRN would be obtained in a change taking place in context of losses (e.g. a change from 5% to 30% of reward probability) compared to a change within a context of gains (e.g. a change from 60% to 85%). Finally, our study suggests that context effects on decision-making are adequately explained by reinforcement learning models. However, it has been suggested that context effects in real life decision-making might not be easily explained by parsimonious learning models [8]. In order to tackle this issue, it would be interesting to consider recent ecologically-valid theoretical models of cognitive control and decision-making that integrate social and phenomenal dimensions with reinforcement learning principles. For instance, Ibanez & Manes [8] suggest that a fronto-insular temporal network would be primarily responsible for how behavioural choices can be constrained by context, including social contexts. In addition, Singer et al., [63] suggest that the insula plays a role in integrating experiential information (“predictive feelings”) into the process of comparing predicted outcomes with actual outcomes.

In conclusion, this study provides evidence that a mere change relative to a prior state of reward contingencies can modulate the electrophysiological distinction between negative and positive outcomes in the FRN time window. This finding suggests that sudden changes relative to prior reward contingencies can constrain neural systems of outcome monitoring. Further, we also found that this pattern of results appeared to be driven at least in part by variations in ERPs to positive outcomes.These findings can potentially contribute to a more mechanistic understanding of emotional phenomena linked to sudden changes between environments, such as negative outlooks caused by life events [1], [2] or the phenomenon of optimism [76]. In both cases, it could be speculated that monitoring systems possibly located mainly in the medial frontal cortex are coding the changes relative to prior circumstances and accordingly re-setting predictions about future events.

### Data Access

In accordance with the policies of PLOS One, the data reported in this article can be accessed upon request addressed to the corresponding authors.

## Supporting Information

Figure S1 **FRN waveforms separated by prior choice.** Averaged ERP waveforms for the FRN plotting ERPs to Wins and Losses separately for prior choice (risk; left, safe, right) and block (PW; top, PL; bottom). Electrode data are taken from the midline fronto-central cluster (MFC) as described in the methods section.
(PDF)

Figure S2 **P3 waveforms separated by prior choice.** Averaged ERP waveforms from the midline parietal (MP) cluster for the Feedback-related P3 plotting ERPs to Wins and Losses separately for prior choice.
(PDF)

Figure S3 **FRN for high and low-risk takers in objective blocks.** Averaged ERP waveforms from the MFC cluster for the FRN plotting ERPs to Wins and Losses separately for high (left) and low (right) risk-takers within the objective reward probability blocks (WD; LD).
(PDF)

Figure S4 **FRN for high and low-risk takers in subjective blocks.** Averaged ERP waveforms from the MFC cluster for the FRN plotting ERPs to Wins and Losses separately for high (left) and low (right) risk-takers within the subjective reward probability blocks (PW; top, PL; bottom).
(PDF)

Figure S5 **FRN for early and late stages of blocks.** Averaged ERP waveforms from the MFC cluster for the FRN plotting ERPs to Wins and Losses separately for early (top) and late (bottom) stages within the PW (left) and PL (right) blocks.
(PDF)

Figure S6 **P3 for early and late stages of blocks.** Averaged ERP waveforms for the P3 plotting ERPs to Wins and Losses separately for early (top) and late (bottom) stages within the PW (left) and PL (right) blocks from the Midline Parietal Cluster.
(PDF)

Figure S7 **Valence specific contrasts.** We used one-tailed t-tests to examine if FRN activity was more negative for unexpected rather than expected contexts. For Wins, both WD-LD and PL-PW contrast were significant (*p*s ≤.05). For Losses, WD-LD was significant (*p*<.05), but not PL-PW (t<1.0). We thank an anonymous reviewer for suggesting this analysis. However, these results have to be considered with caution given that the EVal X EType X Feedback was not significant.
(PDF)

Figure S8 **Attenuation of FRN activity in negative blocks.** We examined the prediction that an attenuation of the FRN might have taken place in negative expectancy contexts. We compared the FRN amplitude, by subtracting Win ERPs from Loss ERPs, in positive, negative and neutral blocks. A one-way ANOVA revealed a significant main effect of context [*F*(1.9, 39.6) = 4.9, *p = *.01, *η* ^2^ = .19] and pairwise comparisons revealed that the FRN difference score for negative contexts was significantly smaller than both positive and neutral contexts (*p*s<.05). In addition, no significant difference was found between positive and neutral contexts (*p*>.40).
(PDF)

Table S1 Frequency of choices across blocks.
(PDF)

Table S2 RTs for choices across blocks.
(PDF)

Table S3 Participant numbers for high risk vs. low risk separation.
(PDF)

Results S1 **Descriptive statistics for the behavioural data.**
(PDF)

Results S2 **FRN and P3 analyses separated by preceding choice (risky vs. safe).**
(PDF)

Results S3 **Individual differences in risk-taking.**
(PDF)

Results S4 **The effects of valence on the FRN by different temporal stages.**
(PDF)
