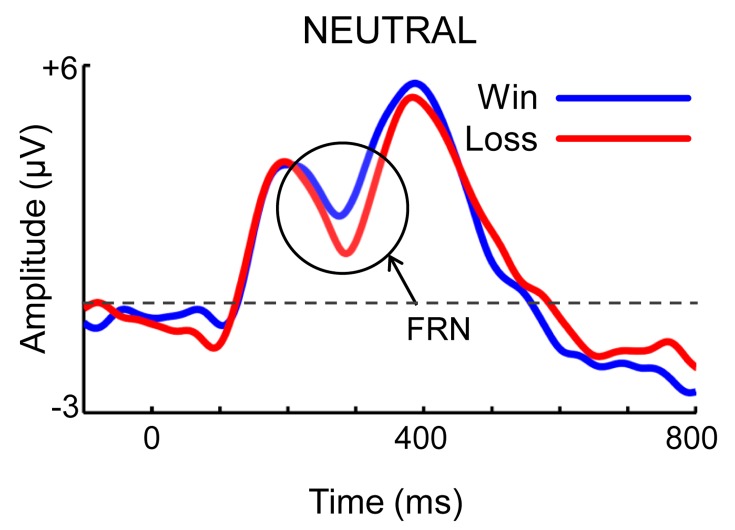# Correction: Relative Changes from Prior Reward Contingencies Can Constrain Brain Correlates of Outcome Monitoring

**DOI:** 10.1371/annotation/f7a918f4-826a-40a7-814b-b150391297bf

**Published:** 2014-01-08

**Authors:** Faisal Mushtaq, Gijsbert Stoet, Amy Rachel Bland, Alexandre Schaefer

Several errors were introduced in the preparation of this manuscript for publication. The text in figures 3, 4 and 5 has been removed or distorted. Please find the correct version of each of the figures here:

Figure 3: 

**Figure:**
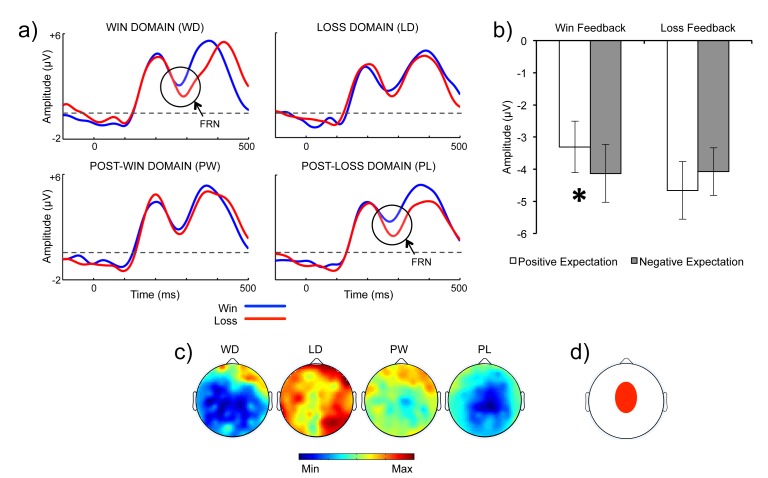


Figure 4: 

**Figure:**
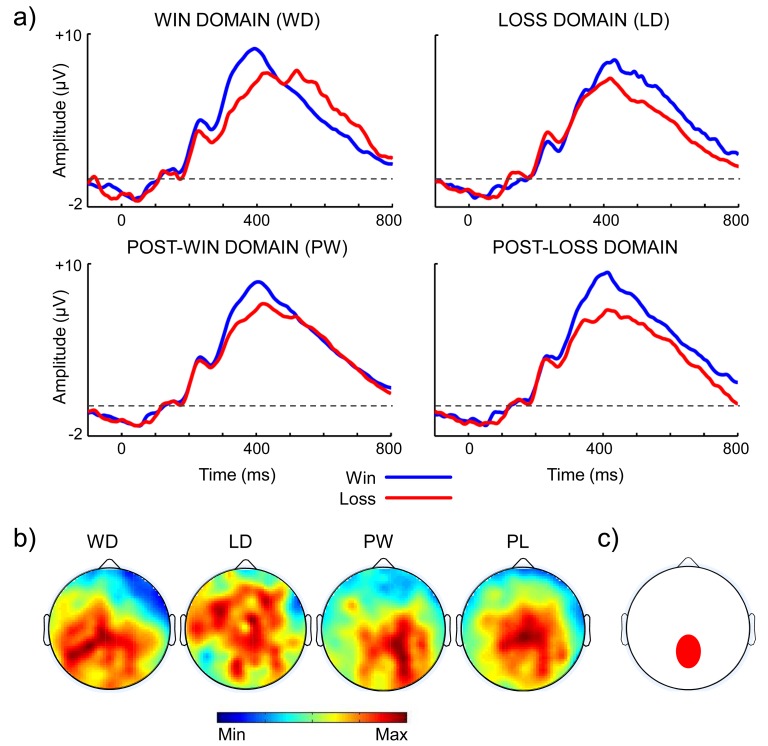


Figure 5: 

**Figure:**