# Supplementary material for: Relative Changes from Prior Reward Contingencies Can Constrain Brain Correlates of Outcome Monitoring
Source: PLoS One. 2013 Jun 20;8(6):e66350. doi: 10.1371/journal.pone.0066350 (PMC3688785; doi:10.1371/journal.pone.0066350)
Supplement: Figure S1 — FRN waveforms separated by prior choice. Averaged ERP waveforms for the FRN plotting ERPs to Wins and Losses separately for prior choice (risk; left, safe, right) and block (PW; top, PL; bottom). Electrode data are taken from the midline fronto-central cluster (MFC) as described in the methods section. (PDF) [file pone.0066350.s001.pdf]

Relative changes from prior reward contingencies can constrain brain correlates of outcome monitoring.

Mushtaq, Stoet, Bland & Schaefer

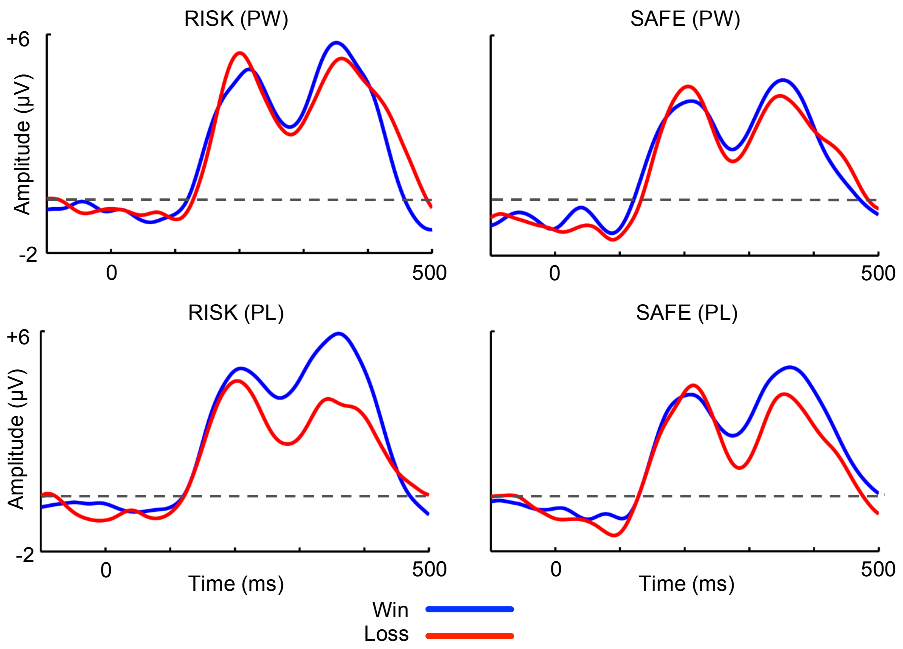

Figure S1- FRN waveforms separated by prior choice. Averaged ERP waveforms for the FRN plotting ERPs to Wins and Losses separately for prior choice (risk; left, safe, right) and block (PW; top, PL; bottom). Electrode data are taken from the midline fronto-central cluster (MFC) as described in the methods section.
