# Supplementary material for: Relative Changes from Prior Reward Contingencies Can Constrain Brain Correlates of Outcome Monitoring
Source: PLoS One. 2013 Jun 20;8(6):e66350. doi: 10.1371/journal.pone.0066350 (PMC3688785; doi:10.1371/journal.pone.0066350)
Supplement: Figure S2 — P3 waveforms separated by prior choice. Averaged ERP waveforms from the midline parietal (MP) cluster for the Feedback-related P3 plotting ERPs to Wins and Losses separately for prior choice. (PDF) [file pone.0066350.s002.pdf]

Mushtaq, Stoet, Bland & Schaefer

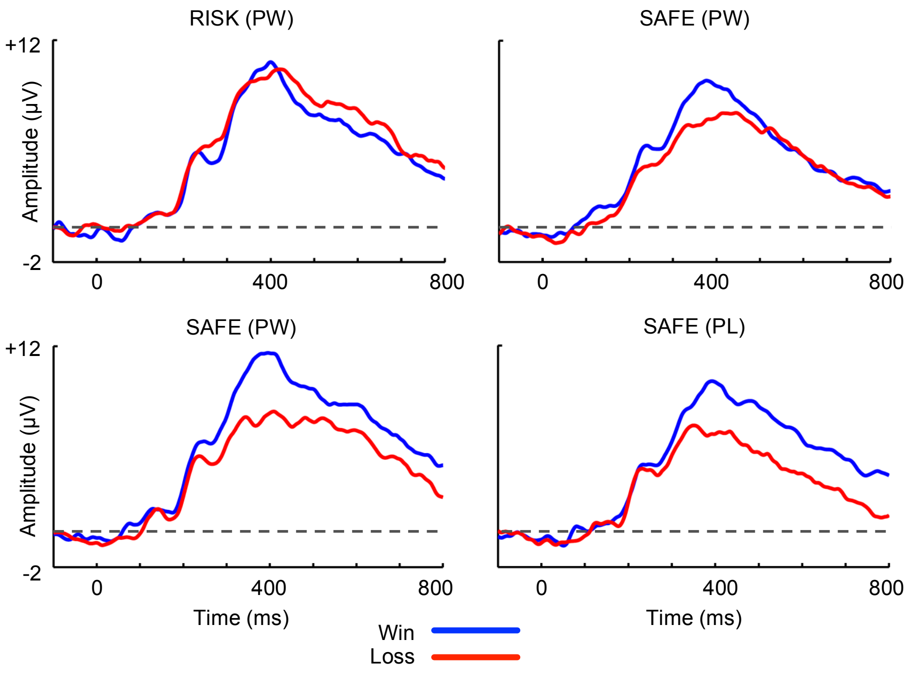

Figure S2- P3 waveforms separated by prior choice. Averaged ERP waveforms from the midline parietal (MP) cluster for the Feedback-related P3 plotting ERPs to Wins and Losses separately for prior choice.
