# Supplementary material for: Relative Changes from Prior Reward Contingencies Can Constrain Brain Correlates of Outcome Monitoring
Source: PLoS One. 2013 Jun 20;8(6):e66350. doi: 10.1371/journal.pone.0066350 (PMC3688785; doi:10.1371/journal.pone.0066350)
Supplement: Figure S4 — FRN for high and low-risk takers in subjective blocks. Averaged ERP waveforms from the MFC cluster for the FRN plotting ERPs to Wins and Losses separately for high (left) and low (right) risk-takers within the subjective reward probability blocks (PW; top, PL; bottom). (PDF) [file pone.0066350.s004.pdf]

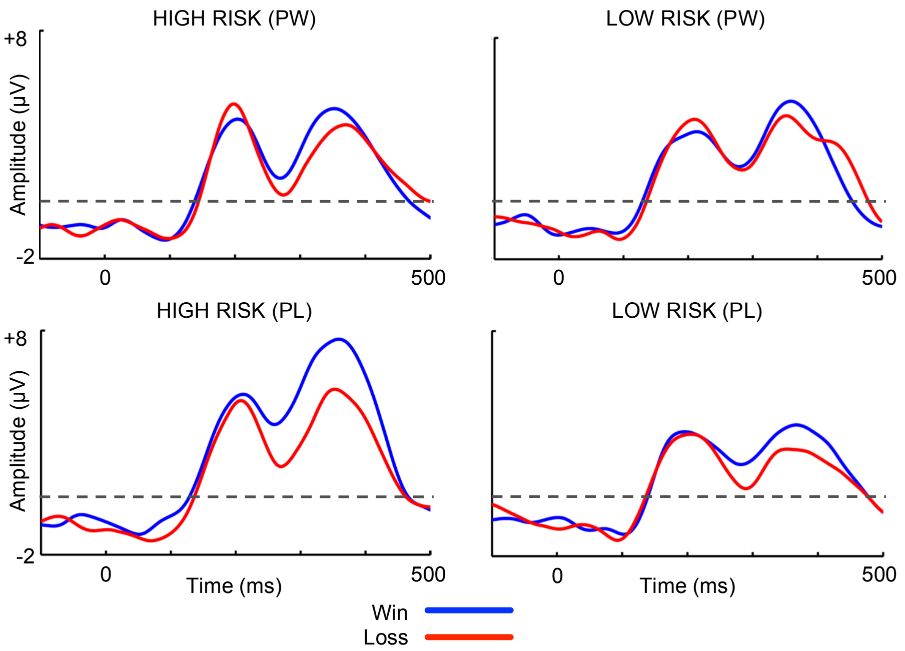

Figure S4- FRN for high and low-risk takers in subjective blocks. Averaged ERP waveforms from the MFC cluster for the FRN plotting ERPs to Wins and Losses separately for high (left) and low (right) risk-takers within the subjective reward probability blocks (PW; top, PL; bottom).
