# Supplementary material for: Relative Changes from Prior Reward Contingencies Can Constrain Brain Correlates of Outcome Monitoring
Source: PLoS One. 2013 Jun 20;8(6):e66350. doi: 10.1371/journal.pone.0066350 (PMC3688785; doi:10.1371/journal.pone.0066350)
Supplement: Figure S5 — FRN for early and late stages of blocks. Averaged ERP waveforms from the MFC cluster for the FRN plotting ERPs to Wins and Losses separately for early (top) and late (bottom) stages within the PW (left) and PL (right) blocks. (PDF) [file pone.0066350.s005.pdf]

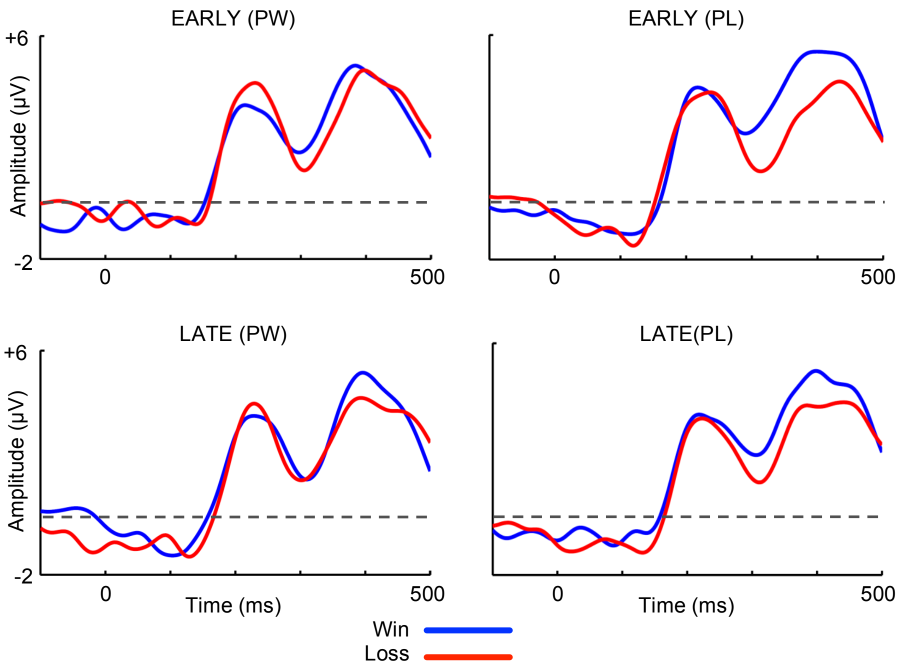

Figure S5- FRN for early and late stages of blocks. Averaged ERP waveforms from the MFC cluster for the FRN plotting ERPs to Wins and Losses separately for early (top) and late (bottom) stages within the PW (left) and PL (right) blocks.
