# Supplementary material for: Relative Changes from Prior Reward Contingencies Can Constrain Brain Correlates of Outcome Monitoring
Source: PLoS One. 2013 Jun 20;8(6):e66350. doi: 10.1371/journal.pone.0066350 (PMC3688785; doi:10.1371/journal.pone.0066350)
Supplement: Figure S6 — P3 for early and late stages of blocks. Averaged ERP waveforms for the P3 plotting ERPs to Wins and Losses separately for early (top) and late (bottom) stages within the PW (left) and PL (right) blocks from the Midline Parietal Cluster. (PDF) [file pone.0066350.s006.pdf]

Relative changes from prior reward contingencies can constrain brain correlates of outcome monitoring.

Mushtaq, Stoet, Bland & Schaefer

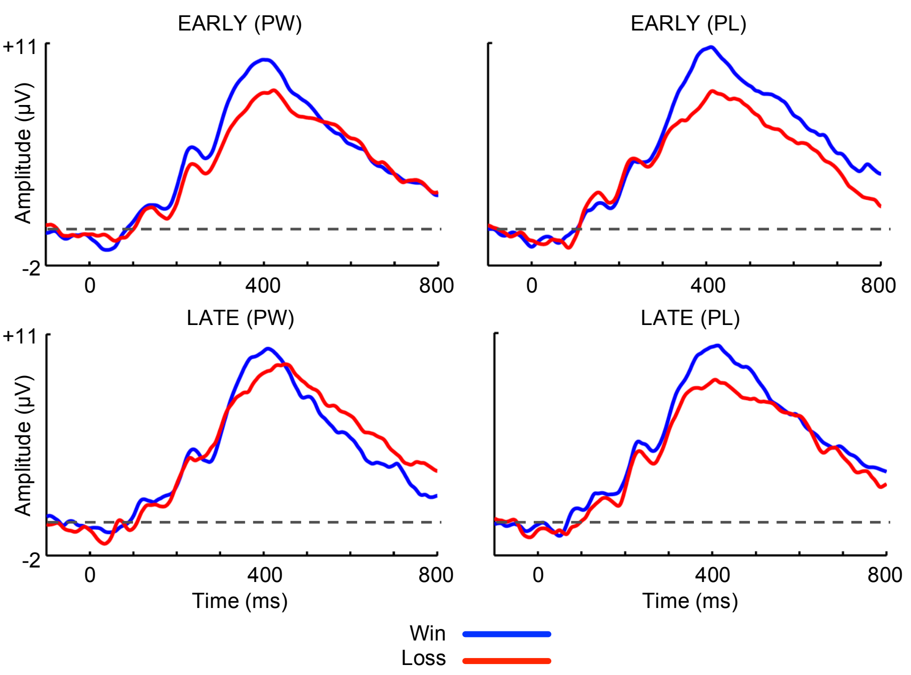


**Figure S6- P3 for early and late stages of blocks.** Averaged ERP waveforms for the P3 plotting ERPs to Wins and Losses separately for early (top) and late (bottom) stages within the PW (left) and PL (right) blocks from the Midline Parietal Cluster.
