# Supplementary material for: Relative Changes from Prior Reward Contingencies Can Constrain Brain Correlates of Outcome Monitoring
Source: PLoS One. 2013 Jun 20;8(6):e66350. doi: 10.1371/journal.pone.0066350 (PMC3688785; doi:10.1371/journal.pone.0066350)
Supplement: Figure S7 — Valence specific contrasts. We used one-tailed t-tests to examine if FRN activity was more negative for unexpected rather than expected contexts. For Wins, both WD-LD and PL-PW contrast were significant (ps ≤.05). For Losses, WD-LD was significant (p<.05), but not PL-PW (t<1.0). We thank an anonymous reviewer for suggesting this analysis. However, these results have to be considered with caution given that the EVal X EType X Feedback was not significant. (PDF) [file pone.0066350.s007.pdf]

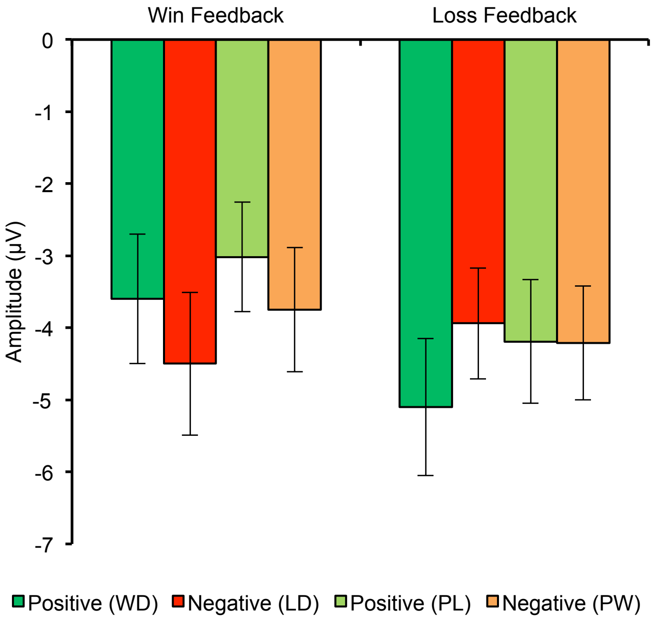

Figure S7- Valence specific contrasts. We used one-tailed t-tests to examine if FRN activity was more negative for unexpected rather than expected contexts. For Wins, both WD-LD and PL-PW contrast were significant (*p*s ≤ .05). For Losses, WD-LD was significant ($p < .05$), but not PL-PW ($t < 1.0$). We thank an anonymous reviewer for suggesting this analysis. However, these results have to be considered with caution given that the EVal X EType X Feedback was not significant.
