# Supplementary material for: Relative Changes from Prior Reward Contingencies Can Constrain Brain Correlates of Outcome Monitoring
Source: PLoS One. 2013 Jun 20;8(6):e66350. doi: 10.1371/journal.pone.0066350 (PMC3688785; doi:10.1371/journal.pone.0066350)
Supplement: Figure S8 — Attenuation of FRN activity in negative blocks. We examined the prediction that an attenuation of the FRN might have taken place in negative expectancy contexts. We compared the FRN amplitude, by subtracting Win ERPs from Loss ERPs, in positive, negative and neutral blocks. A one-way ANOVA revealed a significant main effect of context [F(1.9, 39.6) = 4.9, p = .01, η 2 = .19] and pairwise comparisons revealed that the FRN difference score for negative contexts was significantly smaller than both positive and neutral contexts (ps<.05). In addition, no significant difference was found between positive and neutral contexts (p>.40). (PDF) [file pone.0066350.s008.pdf]

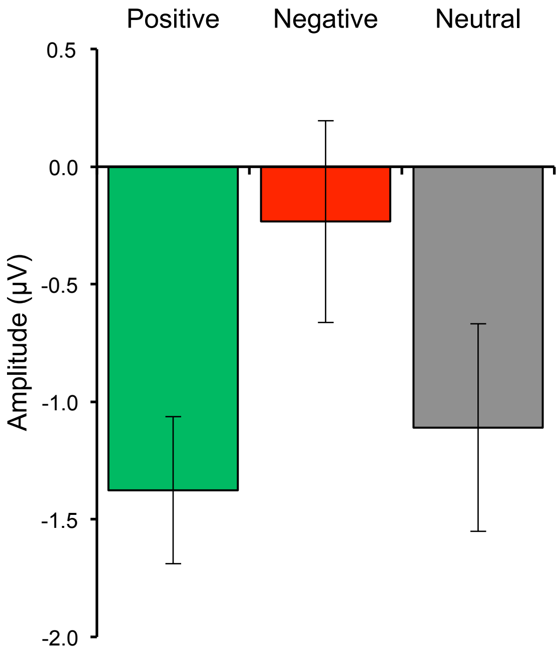

Figure S8- Attenuation of FRN activity in negative blocks. We examined the prediction that an attenuation of the FRN might have taken place in negative expectancy contexts. We compared the FRN amplitude, by subtracting Win ERPs from Loss ERPs, in positive, negative and neutral blocks. A one-way ANOVA revealed a significant main effect of context [$F(1.9, 39.6) = 4.9$, $p = .01$, $\eta^2 = .19$] and pairwise comparisons revealed that the FRN difference score for negative contexts was significantly smaller than both positive and neutral contexts (*p*s < .05). In addition, no significant difference was found between positive and neutral contexts ($p > .40$).
