# Supplementary material for: Relative Changes from Prior Reward Contingencies Can Constrain Brain Correlates of Outcome Monitoring
Source: PLoS One. 2013 Jun 20;8(6):e66350. doi: 10.1371/journal.pone.0066350 (PMC3688785; doi:10.1371/journal.pone.0066350)
Supplement: Table S1 — Frequency of choices across blocks. (PDF) [file pone.0066350.s009.pdf]

Relative changes from prior reward contingencies can constrain brain correlates of outcome monitoring.

Mushtaq, Stoet, Bland & Schaefer

Table S1- Frequency of choices across blocks.

EType	EVal	Choice	Frequency (+/- 1 SEM)
Objective	Positive	Risk	47.23 (2.62)
		Safe	48.09 (2.55)
	Negative	Risk	41.18 (2.73)
		Safe	46.73 (3.11)
Subjective	Positive	Risk	45.32 (2.48)
		Safe	50.27 (2.45)
	Negative	Risk	44.09 (2.56)
		Safe	51.14 (2.47)
