# Supplementary material for: Relative Changes from Prior Reward Contingencies Can Constrain Brain Correlates of Outcome Monitoring
Source: PLoS One. 2013 Jun 20;8(6):e66350. doi: 10.1371/journal.pone.0066350 (PMC3688785; doi:10.1371/journal.pone.0066350)
Supplement: Table S2 — RTs for choices across blocks. (PDF) [file pone.0066350.s010.pdf]

Table S2- RTs for choices across blocks.

EType	EVal	Choice	RT (ms, +/- 1 SEM)
Objective	Positive	Risk	542 (25)
		Safe	568 (30)
	Negative	Risk	557 (24)
		Safe	546 (26)
Subjective	Positive	Risk	572 (25)
		Safe	568 (28)
	Negative	Risk	543 (24)
		Safe	544 (26)
