# Supplementary material for: Relative Changes from Prior Reward Contingencies Can Constrain Brain Correlates of Outcome Monitoring
Source: PLoS One. 2013 Jun 20;8(6):e66350. doi: 10.1371/journal.pone.0066350 (PMC3688785; doi:10.1371/journal.pone.0066350)
Supplement: Table S3 — Participant numbers for high risk vs. low risk separation. (PDF) [file pone.0066350.s011.pdf]

Table S3- Participant numbers for high risk vs. low risk separation.

Block	High Risk	Low Risk
WD	10	12
LD	11	10
PL	8	13
PW	9	12
