# Supplementary material for: Relative Changes from Prior Reward Contingencies Can Constrain Brain Correlates of Outcome Monitoring
Source: PLoS One. 2013 Jun 20;8(6):e66350. doi: 10.1371/journal.pone.0066350 (PMC3688785; doi:10.1371/journal.pone.0066350)
Supplement: Results S1 — Descriptive statistics for the behavioural data. (PDF) [file pone.0066350.s012.pdf]

Results S1- Descriptive statistics for the behavioural data.

We measured the frequency of choices (risky vs. safe) made across blocks (see Table S1) and the response times (RT) of these choices (Table S2). There were no reliable differences in the frequency of choice selection. Specifically, there was no effect of Choice [$F(1,21) = 1.2$, $p = .285$, $\eta^2 = .05$], no EType X Choice interaction [$F(1,21) = 1.5$, $p = .23$, $\eta^2 = .07$], no EVal x Choice [$F(1,21) = 1.0$, $p = .32$, $\eta^2 = .05$] and no EVal X EType X Choice [$F(1,21) = .11$, $p = .74$, $\eta^2 = .01$] interactions. There was a difference in RT data for EVal [$F(1,21) = 4.1$, $p = .056$, $\eta^2 = .16$], reflecting faster responses for negative expectancy valence blocks (*M* = 547±23.7; mean RT±1 standard error of the mean, *SEM*) compared to positive expectancy valence blocks (*M* = 562±24.7). No other effects were observed.
