# Supplementary material for: Relative Changes from Prior Reward Contingencies Can Constrain Brain Correlates of Outcome Monitoring
Source: PLoS One. 2013 Jun 20;8(6):e66350. doi: 10.1371/journal.pone.0066350 (PMC3688785; doi:10.1371/journal.pone.0066350)
Supplement: Results S2 — FRN and P3 analyses separated by preceding choice (risky vs. safe). (PDF) [file pone.0066350.s013.pdf]

Results S2- FRN and P3 analyses separated by preceding choice (risky vs. safe).

For this analysis, WD and LD blocks could not be used, as not enough artifact-free trials were available for a Feedback X Choice design in these blocks. This was expected, given that proportions of wins and loss feedbacks were asymmetrical in these blocks (i.e. only a few losses in WD block and only a few gains in LD block). Therefore, we focused our analyses on PL and PW blocks, where the percentages of gains and losses were similar and enough artifact-free trials (no less than 16 and in average 24 trials per condition) were available for a Feedback X Choice design for 21 participants. In order to verify if the type of choice had any effect on the FRN and P3, we then computed repeated-measures Feedback X Choice ANOVAs separately for PL and PW. We report analyses made on peak-to-peak measures, but the same pattern of results were obtained using absolute peak and mean amplitudes. These results indicate that the effect of valence on FRN activity was not significantly modulated by previous risky vs. safe choice. Regarding P3 data, these analyses confirm that ERPs to reward feedbacks are overall more positive-going than ERPs to nonrewards. It also tentatively suggests that the absence of an effect of valence on PW blocks might be driven mainly by trials preceded by a risky choice.

FRN

PW blocks

There was no main effect of Feedback, [$F(1, 20) = 1.71$, $p = .206$, $\eta^2 = .08$], however, there was a significant main effect of Choice, [$F(1, 20) = 8.85$, $p = .007$, $\eta^2 = .31$], with safe choices (2.54±1.07) more negative going than risk (4.5±.93). There was no Feedback X Choice interaction [$F(1, 20) = 1.06$, $p = .315$, $\eta^2 = .05$].

PL blocks

There was a significant main effect of Feedback, [$F(1, 20) = 9.04$, $p = .007$, $\eta^2 = .31$], with losses (2.27±1.1) more negative going than wins (4.54±1.06). There was also a significant main effect of Choice, [$F(1, 20) = 8.91$, $p = .007$, $\eta^2 = .31$], with safe (2.41±1.1) more negative going than risk (4.41±1.01). There was no Feedback X Choice interaction, [$F(1, 20) = .24$, $p = .63$, $\eta^2 = .01$].
