# Supplementary material for: Relative Changes from Prior Reward Contingencies Can Constrain Brain Correlates of Outcome Monitoring
Source: PLoS One. 2013 Jun 20;8(6):e66350. doi: 10.1371/journal.pone.0066350 (PMC3688785; doi:10.1371/journal.pone.0066350)
Supplement: Results S3 — Individual differences in risk-taking. (PDF) [file pone.0066350.s014.pdf]

Results S3- Individual differences in risk-taking

Here we report analyses based on the suggestion of an anonymous reviewer. The goal of this analysis was to investigate the effects of individual differences in risk-taking on FRN amplitude. We divided participants in two groups: high and low-risk takers within each block. Groups were created by subtracting the total number of safe choices made within a block from the total number of risky choices made within the same block (the risk-seeking score; RSS). A positive RSS meant that the individual made more risky choices than safe (high-risk group) and a negative RSS meant that individuals made more safe choices (low-risk group). Participants who had a 0 RSS within a block were not included in the analysis. It is worth noting that the resulting number of participants in each group was relatively low (see Table S3 for the number of participants assigned to each group within each block). Therefore any conclusions drawn from this data should be taken with caution.

A between subjects ANOVA (Group; High Risk vs. Low Risk X Feedback; Win vs. Loss) was conducted within each block. Peak-to-peak amplitude analyses for the FRN revealed that there was a significant main effect of feedback in WD and PL blocks [F = 13.7, 5.23, p = .001, .03, respectively], but this effect did not reach significance in LD and PW [F = .03, 1.43, p = .865, .26], which is consistent with the FRN results reported in the results section of the manuscript.

Interestingly, there was a significant Group X Feedback interaction in WD [$F(1,20) = 8.34$, $p = .009$, $\eta^2 = .29$], which was driven by a significant main effect of feedback in the low-risk group [$F(1,11) = 22.07$, $p = .001$, $\eta^2 = .67$]. This result showed that loss trials were more negative going (1.29±1.22) than win trials (2.82±1.31). There was no difference in high-risk participants [$F(1,9) < .01$, $p = .99$, $\eta^2 < .01$). The Feedback X Group interaction was not significant in LD [$F(1,19) = 1.16$, $p = .336$, $\eta^2 = .18$], PW [$F(1,20) = .70$, $p = .412$, $\eta^2 = .03$] or PL [$F(2,19) = .52$, $p = .783$, $\eta^2 = .03$] blocks.

These results suggest that overall, individual differences in risk-taking did not modulate the effect of valence on FRN activity for LD, PW and PL blocks. However, a Group X Feedback interaction was found for WD. This specific result can be tentatively interpreted in line with literature suggesting that individuals are risk-averse in the gain domain (Kahneman & Tversky, 1979). However, this conclusion needs to be made with caution given the low count of participants in each risk group. Future research will be needed to explore this question further.

Reference

Kahneman, D., & Tversky, A. (1979). Prospect theory: an analysis of decision under risk. *Econometrica*, *47*, 263.
