# Supplementary material for: Relative Changes from Prior Reward Contingencies Can Constrain Brain Correlates of Outcome Monitoring
Source: PLoS One. 2013 Jun 20;8(6):e66350. doi: 10.1371/journal.pone.0066350 (PMC3688785; doi:10.1371/journal.pone.0066350)
Supplement: Results S4 — The effects of valence on the FRN by different temporal stages. (PDF) [file pone.0066350.s015.pdf]

Results S4- The effects of valence on the FRN by different temporal stages.

The goal of this analysis was to examine if the observed pattern of FRN results was different according to different temporal stages within blocks. We therefore separated each block in two halves (Stage 1 vs. Stage 2). Due to trial number constraints, we could not separate WD and LD blocks. We therefore restricted this analysis to PW and PL blocks, in which sufficient numbers of artefact free trials were available (no less than 16 and in average 22 trials per condition). Peak-to-peak measures for the FRN and P3 are reported, however, equivalent pattern of results were observed using peak and mean amplitude measures.

FRN amplitude

No effect of stage [$F(1,21] < .001$ $p = .985$, $\eta^2 < .001$], Feedback [$F(1,21) = 2.52$, $p = .128$, $\eta^2 = .11$] or a Stage x feedback interaction[$F(1,21) = 2.95$, $p = .10$, $\eta^2 = .12$] were found for the PW block. There was a significant main effect of Feedback [$F(1,21) = 5.62$, $p = .027$, $\eta^2 = .21$] in the PL block, with loss amplitude more negative (-4.83±.63) going than win (-3.64±.53). However, there was no effect of stage [$F(1,21) = .07$, $p = .797$, $\eta^2 < .01$] and no Stage x feedback [$F(1,21) = 1.37$, $p = .255$, $\eta^2 = .06$] interaction. See Figure S5.

P3 amplitude

For the PW block, there was a significant main effect of Feedback [$F(1,21) = 6.93$, $p = .016$, $\eta^2 = .25$], with win trials (9.7±1.09) more positive than loss (8.46±1.11). There was no effect of Stage [$F(1,21) = 1.32$, $p = .262$, $\eta^2 = .06$], and no Stage x Feedback interaction [$F(1,21) = .99$, $p = .332$, $\eta^2 = .05$]. Similarly in the PL block, there was no effect of Stage [$F(1,21) = .19$, $p = .669$, $\eta^2 < .01$], but there was a difference in Feedback amplitude [$F(1,21) = 18.71$, $p < .001$, $\eta^2 = .47$], with wins more positive than losses (7.6±.89). There was no Stage x Feedback interaction [$F(1,21) = .66$, $p = .425$, $\eta^2 = .03$]. See Figure S6.
